# Case Report: Hyperprolinemia type II in a child with autism spectrum disorder and *ALDH4A1* gene variant in a consanguineous family

**DOI:** 10.3389/fped.2025.1726800

**Published:** 2026-01-12

**Authors:** Faisal O. AlQurashi, Bashayer S. Alawam, Bader Alhaddad, Zahra A. Alrebh

**Affiliations:** 1 Department of Pediatrics, College of Medicine, King Fahad Hospital of the University, Imam Abdulrahman Bin Faisal University, Alkhobar, Saudi Arabia; 2 Laboratory Medicine Department, King Fahd Hospital of the University, Imam Abdulrahman Bin Faisal University, Al Khobar, Saudi Arabia; 3 Lifera Omics, Riyadh, Saudi Arabia; 4 Neurodisability and Behavioral Fellow, RCPI Postgraduate Training Program, Royal Collage of Physician of Ireland, Dublin, Ireland; 5 Department of Pediatrics, Qatif Central Hospital, Qatif, Saudi Arabia

**Keywords:** *ALDH4A1*, autism spectrum disorders, global developmental delay, hyperprolinemia type II, proline

## Abstract

Hyperprolinemia type II (HPII) is a rare inherited metabolic disorder caused by the *ALDH4A1* gene variant. Herein, we report a case of a preschool-aged Saudi girl who was born from consanguineous parents and presented with global developmental delay. The patient was clinically diagnosed with autism spectrum disorder with associated disruptive behaviors. Metabolic investigations revealed markedly elevated plasma and urinary proline levels, suggestive of a proline metabolism disorder. Whole-exome sequencing identified a homozygous variant of uncertain significance in the *ALDH4A1* gene, which is associated with autosomal recessive HPII. Genetic testing of the patient's family members showed that all individuals had carrier status with varying zygosity. This case underscores the importance of metabolic and genetic evaluation in children with neurodevelopmental disorders and highlights that HPII can present with a clinical phenotype that overlaps substantially with ASD.

## 1 Introduction

Hereditary hyperprolinemia is divided into two categories: type I (HPI; OMIM # 239500) and type II (HPII; OMIM # 239510), each caused by an autosomal recessive inborn defect of the proline metabolic pathway. HPI is caused by a deficiency in the enzyme proline oxidase (POX), also referred to as proline dehydrogenase (PRODH). The gene responsible for encoding POX, known as *PRODH*, is located on chromosome 22 at the 22q11.21 region. Deletion of 22q11.21 is common in individuals with 22q11.2 microdeletion syndrome, and variations in this gene region have been linked to a higher risk of developing schizophrenia.

HPII is a rare autosomal recessive metabolic disorder caused by a deficiency in the enzyme *Δ*-1-pyrroline-5-carboxylate dehydrogenase (P5CDh). The gene encoding this enzyme, *ALDH4A1*, is located on chromosome 1 at the 1p36.13 region (1, 2). The *ALDH4A1* gene variant leads to impaired proline catabolism and elevated plasma proline levels. Clinically, it is associated with global developmental delay, cognitive impairment, seizures, and behavioral abnormalities (2). Owing to its phenotypic overlap with other neurodevelopmental conditions, HPII diagnosis can be challenging without targeted metabolic evaluation.

A direct link between a defect in proline metabolism and human disease was first reported by Schafer et al. in 1962 (3). The patients in their study exhibited hyperprolinemia alongside cerebral dysfunction, renal abnormalities, hereditary kidney disease, and hearing loss. Since then, numerous cases of hyperprolinemia have been documented, revealing a range of clinical presentations, including asymptomatic cases within affected families (4–13). Some researchers have questioned whether these clinical features are coincidentally associated with hyperprolinemia (9). Knowledge of the connections between proline metabolism, its physiological roles, and related diseases has significantly expanded in recent years. To date, at least six enzymes, three transport proteins, and seven structural genes have been identified as key players in the metabolic pathways involving proline and its immediate derivatives. Their biochemical properties, tissue distribution, regulatory mechanisms, subcellular localization, and, in some cases, structural details have been well characterized (14). Despite this progress, the precise clinical manifestations and prevalence rates of HPI and HPII remain unclear.

Here, we describe the case of a girl aged 5 years and 5 months, presenting with global developmental delay, significant speech and language deficits, and atypical social behaviors consistent with autism spectrum disorder (ASD). A history of developmental regression and a family history of neurodevelopmental disorders raised further suspicion for an underlying metabolic etiology. Biochemical and genetic testing confirmed a diagnosis of HPII. This case demonstrates the importance of metabolic screening in children with unexplained developmental and behavioral abnormalities, highlighting the role of early recognition and multidisciplinary management in improving outcomes.

## 2 Case description

A preschool-aged Saudi girl of consanguineous parents presented to our hospital with parental concerns regarding speech and language delay, atypical behaviors, and a history of developmental regression. The parents began to be concerned when the patient was 2.5 years old, following loss of previously acquired social skills, including reduced eye contact and social initiation skills. The patient's developmental history was remarkable for global developmental delay, affecting the fine motor, social/emotional, speech/language, and cognitive/self-help domains. However, gross motor skills were preserved, with no concerns reported. Fine motor skills were mildly delayed; while she could perform basic tasks (e.g., using a mature pincer grasp, handling light switches and doorknobs), she struggled with utensils, buttons, and zippers. Speech and language development was significantly delayed as she was nonverbal, producing only gibberish sounds and not using or understanding gestures. She exhibited inconsistent responses to her name and limited ability to follow one-step commands. Echolalia was noted, primarily imitating song intonations. Cognitive abilities were similarly delayed, with challenges in recognition of body parts and limited engagement in functional or symbolic play.

**Figure 1:**
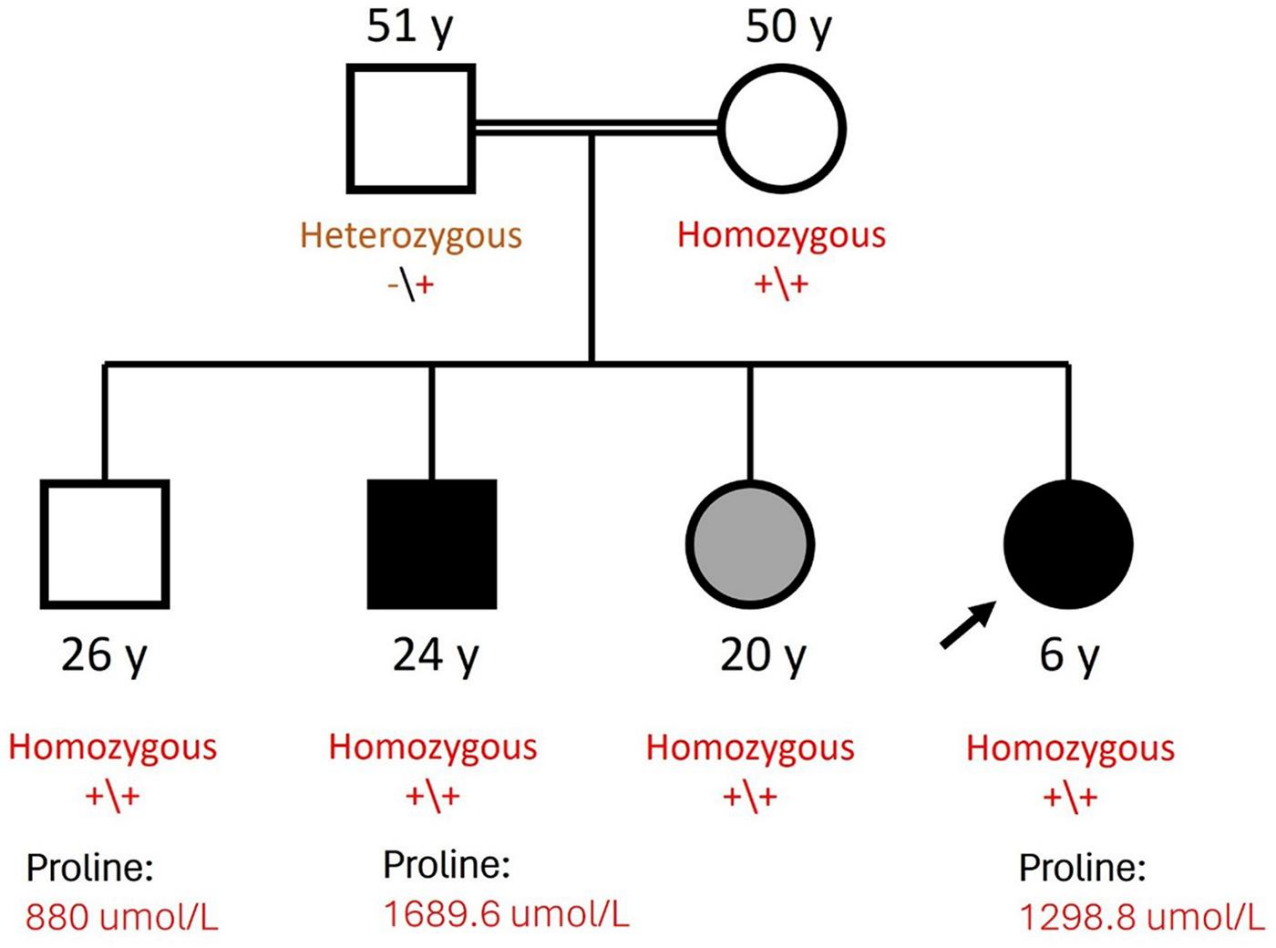
Family pedigree.

Socially, she preferred solitary play and demonstrated an awkward social approach, often limited to physical activities. She lacked social sharing, joint attention, and emotional reciprocity. She did not understand her parents’ feelings; however, she may have inconsistently understood others’ facial expressions. Her nonverbal communication skills, including pointing and gesturing, were either limited or absent. Her family history was notable for consanguinity of the parents, who are first-degree cousins. The mother, aged 50 years, was a housewife with hypertension but no reported neurological or psychiatric concerns. The father, aged 51 years, had hypertension and diabetes mellitus and a history of learning difficulties during school years. From the paternal side, we noted a history of undiagnosed autism traits in the grandfather and a nephew with ASD and attention deficit/hyperactivity disorder. The patient had three siblings: a 26-year-old healthy brother; a 24-year-old brother with ASD, obsessive–compulsive disorder (OCD), and depression; and a 20-year-old sister with hydrocephalus (Figure 1).

On physical examination, the patient's initial vital signs were as follows: oxygen saturation, 99% on room air; temperature, 36.2 °C; pulse rate, 91 beats per minute; respiratory rate, 22 breaths per minute; and blood pressure, 110/76 mmHg. Regarding growth parameters, her height was 112 cm (25th percentile for age and sex); weight, 17 kg (10th percentile for age and sex); body mass index, 13.55 kg/m^2^ (above the 5th percentile for age and sex); and head circumference, 49.5 cm (25th percentile for age and sex). Systemic physical examination revealed generalized hypotonia and hyporeflexia. No neurocutaneous stigmata, dysmorphic features, or signs of scoliosis were noted. The cranial nerves were intact, and her gait was normal.

Based on thorough assessment and clinical observation, the patient met the Diagnostic and Statistical Manual of Mental Disorders, 5th edition criteria for ASD. Assessment via applying Autism Diagnostic Observation Schedule-2 (ADOS-2) tool indicated high level of autism spectrum-related symptoms. Her ASD diagnostic profile was associated with language impairment and possible intellectual impairment. Additionally, the patient exhibited disruptive and explosive behaviors, including daily verbal outbursts (shouting, screaming) and occasional self-harm (self-scratching, head banging). These behaviors were mainly triggered by unmet expectations or changes in routine.

## 3 Diagnostic assessments

Neuroimaging via brain magnetic resonance imaging was unremarkable. Initial metabolic testing via plasma amino acid analysis by the National Reference Laboratory revealed low cystine (5.2 μmol/L, reference range: 9.8–29.2 μmol/L), high ornithine (101.6 μmol/L, reference range: 27.7–91.2 μmol/L), and very high proline (1,298.8 μmol/L, reference range: 84.5–365.0 μmol/L) levels. Urine organic acid analysis by Eurofins Biomnis Laboratories revealed the presence of N-(pyrrole-2-carboxyl) glycine. Repeat plasma amino acid analysis a year after the first analysis by Bioscientia International Laboratories revealed increasing proline levels (2,120 μmol/L), high serine (193 μmol/L, reference range: 69.0–187 μmol/L), high alanine (615 μmol/L, reference range: 152–547 μmol/L), and high tyrosine (130 μmol/L, reference range: 24.0–115 μmol/L) levels. Urine amino acid analysis by Bioscientia International Laboratories revealed high aspartic acid (33 μM/mM Crea, reference range: 2.0–8.0 μM/mM Crea), high hydroxyproline (178 μM/mM Crea, reference range: <13 μM/mM Crea), high serine (109 μM/mM Crea, reference range: 38.0–93.0 μM/mM Crea), very high proline (2,893 μM/mM Crea, reference range: <6 μM/mM Crea), very high glycine (1,552 μM/mM Crea, reference range: 91.0–246.0 μM/mM Crea), and high alpha aminobutyric acid (9 μM/mM Crea, reference range: <5 μM/mM Crea) levels, with a creatinine level of 1,047 mg/L.

All other basic laboratory parameters (complete blood count, renal function, liver function, creatine kinase, iron profile, thyroid profile) were within normal ranges. Diagnostic exome sequencing was performed at CENTOGENE GmbH as described previously (15). All rare variants with a minor allele frequency lower than <1% and predicted moderate/high impact on protein function were evaluated. A novel homozygous missense variant was identified in the *ALDH4A1* gene, NM_003748.3:c.1252G>C (p.Gly418Arg) (GRCh37/hg19), which has not been reported before and presents in gnomAD 2.1.1 only in the heterozygous state. *In silico* prediction tools (REVEL, CADD, and SIFT) supported its pathogenic effect. Segregation analysis through targeted Sanger sequencing revealed that almost all the family members had the variant in the homozygous state; the father alone was found to carry the variant in the heterozygous state. This prompted us to further investigate the proline level in the patient's asymptomatic, homozygotic brother; surprisingly, he was found to have high plasma protein levels (Table 1). Following the ACMG guidelines, the variant is classified as a variant of uncertain significance (VUS) based on the criteria: PP3_Strong, PM2_Supporting, BS4_Strong, and BP1_Supporting.

**Table 1:** Family genetic testing results.

Subject (age)	Gene	Variant coordinates	Amino acid change	Zygosity	Phenotype	Remarks
Index case (5.5 years old)	*ALDH4A1*	NM_003748.3:c.1252G>C	pGly418Arg	homozygous	ASD, with language and intellectual impairment	High proline (plasma: 1,298.8 µmol/L, urine: 2,893 μM/mM Crea)
Sister (20 years old)	*ALDH4A1*	NM_003748.3:c.1252G>C	pGly418Arg	Homozygous	Hydrocephalus	N/A
Brother (24 years old)	*ALDH4A1*	NM_003748.3:c.1252G>C	pGly418Arg	Homozygous	ASD, OCD, depression	High proline (plasma 1,689.6 µmol/L) Urine organic acid [presence of N-(pyrrole-2-carboxyl)glycine]
Brother (26 years old)	*ALDH4A1*	NM_003748.3:c.1252G>C	pGly418Arg	Homozygous	Asymptomatic	High proline (plasma: 880 µmol/L)
Mother (50 years old)	*ALDH4A1*	NM_003748.3:c.1252G>C	pGly418Arg	Homozygous	Asymptomatic	N/A
Father (51 years old)	*ALDH4A1*	NM_003748.3:c.1252G>C	pGly418Arg	Heterozygous	Asymptomatic	N/A

### 3.1 Outcome and follow-up

The patient has been enrolled in early intervention programs since the age of 3 years, attending a specialized daycare with speech-language therapy, occupational therapy, and applied behavioral analysis sessions. Improvements in eye contact, responsiveness to her name, and adaptive functioning have been noted. Although the patient was started on a course of vitamin B6 (500 mg/day), no effects were observed. Formal psychometric assessment to determine accurate cognitive and adaptive functioning levels has been scheduled. No adverse and unanticipated events were reported by family during the follow-up period.

## 4 Discussion

l-proline concentrations in tissues and body fluids is largely mediated by the interaction between two key enzymes: POX and pyrroline-5-carboxylate (P5C) reductase. P5C serves as a central intermediate in this process; genetic disorders that affect proline metabolism typically arise from defects in enzymes linked to P5C. HPI and HPII are distinguished by specific biochemical and genetic defects in the proline degradation pathway. HPI results from a deficiency in POX, an enzyme located in the inner mitochondrial membrane that initiates proline breakdown by converting it to P5C. The next step in this pathway is the transformation of P5C into glutamate by P5CDh, a nicotinamide adenine dinucleotide (+)-dependent mitochondrial matrix enzyme. A deficiency in P5CDh leads to HPII, an autosomal recessive condition characterized by elevated levels of both proline and P5C (1, 2).

HPII is diagnosed by identifying significantly elevated plasma proline levels (10–15 times normal levels) and the accumulation of P5C (2). Biochemical assays, such as amino acid chromatography, are used to detect these abnormalities. Genetic testing can confirm pathogenic variants in the *ALDH4A1* gene, responsible for P5CDh deficiency. Urinary amino acid profiling and enzymatic activity assays in fibroblasts may provide further confirmation (14). Neuroimaging and renal function tests may help assess associated complications in symptomatic cases. In the present case, plasma and urine proline levels were significantly elevated, and the *ALDH4A1* gene variant was genetically confirmed. Clinically, no seizures or significant neuroimaging findings were present; however, the diagnostic criteria for ASD were met. The absence of seizures and positive neurological findings on physical examination contradicts the previously reported phenotypes that highlight an organic neurological manifestation of the gene variant (12). Of note, ASD is a genetically heterogeneous condition, and our findings do not establish *ALDH4A1* as an autism susceptibility gene. Rather, they indicate that the metabolic dysregulation in HPII can result in a neurobehavioral profile that may meet diagnostic criteria for ASD, representing one of many potential biological pathways leading to this phenotype (16).

The clinical presentation of HPII is highly variable, making it challenging to identify HPII based on symptoms alone. Although some patients may experience developmental delays, seizures, or encephalopathy of unknown etiology, others may present with minimal or no neurological symptoms. Owing to this variability, HPII cannot be diagnosed solely on a clinical basis. In patients presenting with unexplained neurological symptoms, such as persistent seizures or developmental delay, plasma amino acid analysis should be considered. HPII is characterized by markedly elevated plasma proline levels, often exceeding 1,500 μmol/L, which is significantly higher than the levels seen in HPI. However, secondary causes, such as lactic acidosis, must be ruled out because they can also result in elevated proline levels. HPII diagnosis is confirmed by the detection of P5C in urine using gas chromatography–mass spectrometry. Enzyme activity assays using cultured lymphocytes, fibroblasts, and leukocytes can also help evaluate P5CDh involvement. Additionally, genetic testing is feasible, as the causative gene (*ALDH4A1*) has been cloned, and various gene variants have been documented (2).

Based on further genetic testing of the patient's family members, the *ALDH4A1* gene variant was identified in three asymptomatic members, including both parents and one brother who exhibited high levels of serum proline. Similar findings were previously found during a regular serum amino acid screening program conducted in Sicily in 1997, with three siblings who were clinically normal but were found to have HPII (9); although both parents’ blood proline and glycine concentrations were normal, all three children displayed noticeable hyperglycemia in addition to the basic biochemical characteristics of HPII. The clinical normalcy of patients with hyperprolinemia may indicate that type I and II metabolic diseases are benign. Our pedigree further underscores marked phenotypic heterogeneity in HPII: despite shared familial genetics, affected individuals can demonstrate divergent clinical trajectories. In this family, the same ALDH4A1 variant is observed in individuals with neurodevelopmental/neuropsychiatric phenotypes and markedly elevated proline levels, as well as in relatives who remain clinically asymptomatic despite biochemical abnormalities. This pattern is consistent with incomplete penetrance and variable expressivity, and it raises the possibility of additional modifiers such as age-related factors, intercurrent environmental exposures (e.g., infection-related stressors), and/or interacting genetic background that may influence clinical expression. Accordingly, biochemical and genetic results should be interpreted in conjunction with longitudinal clinical assessment, particularly when cascade testing identifies asymptomatic relatives with persistent hyperprolinemia.

Geraghty et al. (11) conducted a genetic analysis of the *ALDH4A1* gene in four individuals from a family diagnosed with HPII, identifying four distinct gene variants. Two of these were frameshift gene variants: a single-base deletion of guanine at nucleotide 21 (A7fs(-1)) and a single-base insertion of thymine after nucleotide 1,563 (G521fs(+1)). The remaining two were missense gene variants: S352L and P16L. Notably, the G521fs(+1) gene variant has become a defining genetic marker for the HPII phenotype.

Furthermore, Flynn et al. (17) identified HPII in a large family of Irish travelers, a community characterized by consanguinity and high birth rates. Among the 312 individuals from 71 interconnected families, 13 individuals had confirmed HPII, and 50 individuals had mild hyperprolinemia. The G521fs(+1) gene variant was also identified. This study was the first to describe the molecular genetic basis of this metabolic disorder (17). Importantly, their analysis revealed a strong correlation between HPII and childhood seizures, although no consistent link was found between HPII and cognitive impairment; most adults diagnosed with HPII are in good health (17). With respect to treatment and prognosis, HPII seizures often emerge during childhood and are frequently triggered by infections. Effective seizure management is essential to prevent complications, such as febrile seizures and status epilepticus. Unlike in other metabolic disorders, dietary interventions do not significantly reduce plasma proline levels in HPII. A key factor contributing to seizure activity is the accumulation of P5C, which inactivates vitamin B6, a critical cofactor in several neurological processes. This mechanism is supported by pediatric cases of HPII-related seizures with normal nutrition but with functional vitamin B6 deficiency. In such cases, the P5C-mediated inactivation of pyridoxine may play a central role. Therefore, and according to the International PDE Consortium clinical practice guidelines, long-term vitamin B6 supplementation (children, adolescents, and adults: 30 mg/kg/day with a maximum of 500 mg/day) could be beneficial in preventing seizure recurrence (18, 19). In the current case, a trial of vitamin B6 supplementation in the absence of seizures did not exhibit any clinical or behavioral benefits (20).

Regarding management recommendations and long-term follow-up, there are no universally standardized management guidelines for HPII; therefore, care is individualized and is typically centered on anticipatory guidance and symptom-directed treatment. Given that childhood seizures in HPII have been reported to be infection-triggered, families should receive counseling on seizure recognition and a clear plan for urgent assessment during febrile illnesses. Where seizures occur or where functional pyridoxine depletion is suspected, vitamin B6 supplementation may be considered in view of the proposed P5C-mediated inactivation of vitamin B6–dependent pathways. In parallel, patients should receive structured neurodevelopmental follow-up and early intervention/behavioral therapies when indicated, as implemented in the index case. For asymptomatic relatives identified through cascade testing who demonstrate persistent biochemical abnormalities (e.g., elevated proline), we recommend periodic clinical surveillance focusing on neurological symptoms (particularly seizures) and neurodevelopment/behavioral concerns, with follow-up tailored to age and clinical trajectory.

Additionally, recent animal studies have identified oxidative stress in the brain tissues of rats with elevated proline levels, suggesting a potential therapeutic role for antioxidants, such as vitamins E and C, as well as glutathione. Some authors recommend early introduction of these agents to mitigate oxidative damage (13). While acute symptoms, such as seizures, can be managed effectively, allowing for generally favorable outcomes and normal development, the long-term neurological prognosis remains uncertain. Given the potential for behavioral challenges, individuals with HPII should undergo regular monitoring and follow-up (21).

In conclusion, this case highlights the variable expressivity of HPII, especially the cognitive and behavioral profiles. It also demonstrates the importance of comprehensive metabolic and genetic evaluations in children with neurodevelopmental disorders, along with work-up for potential phenotypic overlap between HPII and ASD.

## 5 Patient perspective

Following identification of hyperprolinemia type II and the associated ALDH4A1 variant, the patient's caregivers emphasized that establishing an etiologic diagnosis was valuable for clarifying prognosis, guiding follow-up planning, and supporting informed decisions regarding ongoing developmental and behavioral interventions. The family reported high interest in cascade evaluation within the extended family, with the intent of identifying relatives with similar neurodevelopmental concerns and facilitating timely medical assessment and appropriate investigations. In view of the logistical demands of coordinating multidisciplinary appointments and diagnostic testing, social working services were engaged to provide funding support and logistical facilitation, which the family considered essential for ensuring continuity of care and access to recommended services.
